# Mediterranean diet adherence and risk of colorectal cancer: the prospective Netherlands Cohort Study

**DOI:** 10.1007/s10654-019-00549-8

**Published:** 2019-09-07

**Authors:** Maya Schulpen, Piet A. van den Brandt

**Affiliations:** 1 Department of Epidemiology, GROW-School for Oncology and Developmental Biology, Maastricht University Medical Centre, P.O. Box 616, 6200 MD Maastricht, The Netherlands; 2 Department of Epidemiology, CAPHRI-School for Public Health and Primary Care, Maastricht University Medical Centre, Maastricht, The Netherlands

**Keywords:** Mediterranean diet, Colorectal cancer, Subsites, Cohort study, Epidemiology, Prevention

## Abstract

Mediterranean diet (MD) adherence has been associated with a large variety of health benefits. However, prospective studies investigating the relation between MD adherence and colorectal cancer risk had inconsistent results. In this analysis of the Netherlands Cohort Study (NLCS), we evaluated sex- and subsite-specific associations of MD adherence with colorectal cancer risk. In 1986, 120,852 subjects filled out the NLCS baseline questionnaire, which incorporated a 150-item food frequency questionnaire. MD adherence was estimated through alternate Mediterranean diet scores including and excluding alcohol (aMED and aMEDr, respectively). Using 20.3 year follow-up data, 1993 male and 1574 female colorectal cancer cases could be included in multivariable case-cohort analyses. aMEDr was not significantly associated with colorectal cancer risk, regardless of sex. Hazard ratios (95% confidence intervals) per two-point increment were 1.04 (0.95–1.13) for men and 0.97 (0.88–1.07) for women. Additionally, there was no evidence of an inverse association with any of the colorectal cancer subsites (colon, proximal colon, distal colon, and rectum). In women, the association between aMEDr and colorectal cancer risk was significantly modified by smoking status (*P*_interaction_ = 0.015). Comparable results were obtained for the original aMED including alcohol. In conclusion, higher MD adherence was not associated with a reduced risk of colorectal cancer or anatomical subsites in the context of a Dutch population.

**Electronic supplementary material:**

The online version of this article (10.1007/s10654-019-00549-8) contains supplementary material, which is available to authorized users.

## Introduction

Globally, colorectal cancer was an important contributor to the total cancer burden in 2018, ranking third and second in terms of incidence and mortality, respectively [1]. The global burden of colorectal cancer is expected to increase even further in the next decade. In 2030, over 2.2 million people are estimated to be diagnosed with colorectal cancer, whereas more than 1.1 million people are expected to die from this disease [2]. Colorectal cancer is a slow-growing disease [3], which offers the opportunity to intervene during the disease development process using preventive measures. These preventive strategies could for instance focus on maintenance of a healthy diet.

The traditional Mediterranean diet (MD), typical for the olive-cultivating areas bordering the Mediterranean basin in the early 1960s, has been associated with a large variety of health benefits, including decreases in all-cause mortality as well as cardiovascular disease risk and mortality [4–8]. This dietary pattern is characterized by the consumption of large quantities of vegetables, legumes, fruits, nuts, whole grains, and olive oil (rich in monounsaturated fatty acids, MUFA). In contrast, intakes of foods from animal origin (e.g. dairy and meat) are low. Finally, wine is consumed in moderate amounts, particularly during meals [4, 5].

The relation between a priori defined MD adherence and colorectal cancer risk has been evaluated in a number of prospective studies so far, with mixed results. Though some studies reported MD adherence to be associated with a significantly reduced colorectal cancer risk [9–12], inverse associations were absent or only observed in specific subgroups in others [13–20]. Additionally, heterogeneity of associations across the sexes and colorectal cancer subsites was indicated [9, 10, 12, 13, 16, 17, 20].

The colorectum can anatomically be divided in the proximal colon, distal colon, and rectum. Depending on the anatomical subsite, colorectal tumors may develop through distinct molecular pathways and show varying patterns of (epi)genetic changes [21, 22]. Furthermore, differences have been shown in subsite-specific incidence trends and survival [21, 22]. Because of their potentially distinct etiologies, cancers of the proximal colon, distal colon, and rectum should initially be considered as separate endpoints in epidemiological studies.

In the present analysis, we aimed to investigate associations of MD adherence with risks of colorectal cancer and anatomical subsites (colon, proximal colon, distal colon, and rectum) in the prospective Netherlands Cohort Study (NLCS). The level of MD adherence was assessed using a priori defined MD scores with and without alcohol component. Moreover, associations were estimated separately for men and women.

## Methods

### Study population and cancer follow-up

The NLCS was conducted among 58,279 men and 62,573 women, who were aged 55–69 years [23–26]. At baseline (September 1986), information on diet and other cancer risk factors was gathered via a self-administered questionnaire. Data were processed and analysed using the case-cohort method, in which cases are derived from the entire cohort and person-years at risk are estimated based on a subcohort. Therefore, a random subcohort (N = 5000) was selected immediately after baseline and vital status information of subcohort members was acquired biennially [23, 26, 27]. Follow-up for cancer incidence was accomplished via annual record linkage with the Netherlands Cancer Registry and the nationwide Dutch Pathology Registry (PALGA) [24]. The NLCS was approved by institutional review boards from Maastricht University and the Netherlands Organization for Applied Scientific Research.

**Fig. 1:**
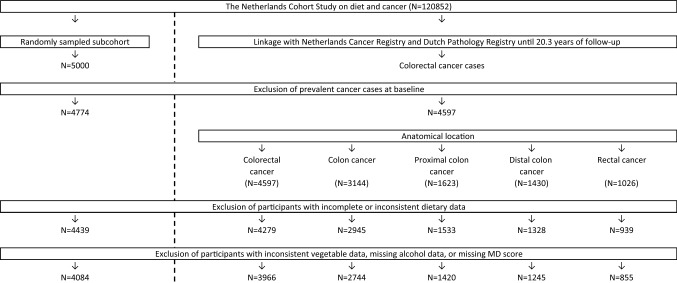
Flow diagram of the number of participants of the Netherlands Cohort Study, who are eligible for inclusion in the analyses concerning colorectal cancer (case-cohort design). *MD* Mediterranean diet

After 20.3 years of follow-up, 4084 subcohort members and 3966 cases with incident and microscopically confirmed colorectal cancer (ICD-O-3 codes: C18–C20) were eligible for inclusion in the present analyses (Fig. 1). Eligible study participants did not have a history of cancer at baseline (except skin cancer), had complete and consistent dietary data, and had data available on alcohol consumption and MD adherence.

### Exposure assessment

Habitual dietary intake during the year preceding baseline was assessed using a 150-item, semi-quantitative food frequency questionnaire (FFQ) [25, 28]. Previously, it has been shown that this FFQ performed adequately and that dietary habits were reproducible for over at least 5 years [25, 28]. The 1986 Dutch food composition (NEVO) table was used to calculate nutrient intakes from the FFQ data [29].

### Mediterranean diet adherence

MD adherence was assessed using the alternate Mediterranean diet score (aMED), which is a variant of the traditional Mediterranean diet score (tMED) developed by Trichopoulou et al., that was adapted for usage in the United States [30–33]. aMED assesses relative MD adherence based on energy-adjusted mean daily intakes of nine food groups with typically high or low consumption in the MD [32, 33]. Each food group is scored by 0 or 1 points, creating a sum score with a maximum value of 9 points (highest level of MD adherence). Subjects receive 1 point for: high intakes (≥ sex-specific median) of vegetables (excluding potatoes), legumes, fruits, nuts, whole grains, and fish; a high (≥ sex-specific median) MUFA to saturated fatty acid (SFA) ratio; a moderate alcohol intake (5–25 g/day); and a low intake (< sex-specific median) of red and processed meats [32, 33].

Moderate and heavy alcohol consumption have been associated with an increased colorectal cancer risk [34, 35]. Therefore, MD adherence was also assessed using a reduced variant of aMED (aMEDr) that does not include alcohol and ranges from 0 to 8 points. Because of the positive association between alcohol consumption and colorectal cancer risk, we will concentrate on results obtained using aMEDr in this article. MD score values were grouped into three MD adherence categories [low (0–3), middle (4–5), and high (6–8 (9))] and were continuously modelled per two-point increment [33].

### Statistical analyses

Cox proportional hazards analyses were conducted to estimate hazards ratios (HRs) and 95% confidence intervals (95% CIs) for sex-specific associations of MD adherence with incidences of total colorectal cancer and anatomical subsites (colon, proximal colon, distal colon, and rectum). Duration of follow-up was used as time variable and person-years at risk of subcohort members were calculated from baseline until colorectal cancer diagnosis, death, emigration, loss to follow-up, or end of follow-up, whichever came first. To account for the increased variance inherent to the case-cohort design, we estimated standard errors using the robust Huber–White sandwich estimator [36]. Scaled Schoenfeld residuals tests and –ln(–ln) survival plots confirmed that it was appropriate to assume proportionality of hazards for the exposure variables [37].

MD scores were included as categorical and continuous terms in age- and multivariable-adjusted Cox models. Based on the literature, we included the following predefined confounders in the multivariable-adjusted models: age at baseline, cigarette smoking behaviour (status, frequency, and duration), body mass index (BMI), alcohol consumption (except for models containing the original aMED including alcohol), total daily energy intake, highest level of education, non-occupational physical activity, and family history of colorectal cancer. Other covariates considered were height, history of diabetes, history of chronic bowel irritation, use of hormone replacement therapy (women only), and long-term use of non-steroidal anti-inflammatory drugs. These factors did not change the HR estimates of aMEDr ≥ 10% and were therefore not included in the final model.

*P* values for trends over the MD adherence categories were obtained by appointing sex-specific median MD score values among subcohort members to each category and fitting these as continuous terms in Cox regression models. Performances of models including MD score variants with and without alcohol (aMED and aMEDr, respectively) were compared using Akaike’s Information Criterion (AIC) [38]. Statistical significance of differences in associations with aMEDr across the anatomical locations of colorectal cancer (colon, proximal colon, distal colon, and rectum) was tested using a competing risks procedure, by which standard errors were estimated using a bootstrapping method developed for the case-cohort design [39, 40].

Stratified analyses were performed to evaluate associations of aMEDr with colorectal cancer risk across levels of cigarette smoking status, alcohol consumption, BMI, educational level, and family history of colorectal cancer. Interaction terms between aMEDr and these potential effect modifiers were added to the models to test the statistical significance of potential differences. To test the sensitivity of our results, analyses were repeated excluding the first 2 years of follow-up. Furthermore, the total follow-up time was divided into three periods (≤ 2, > 2 to ≤ 10, and > 10 years).

As an additional sensitivity analysis, we compared the population-dependent aMED to the absolute WCRF/AICR diet score, which is based on the dietary recommendations for cancer prevention issued by the World Cancer Research Fund/American Institute for Cancer Research (WCRF/AICR) in 2007 [41]. Our WCRF/AICR diet score is based on the WCRF/AICR score developed in the European Prospective Investigation into Cancer and Nutrition (EPIC) cohort [42, 43] and operationalizes the recommendations concerning foods and drinks that promote weight gain, plant foods, red and processed meats, (alcohol), and salt. A detailed description of the calculation of the score has been published previously [44]. Score variants were created including and excluding the alcohol recommendation, resulting in sum scores ranging from 0 to 4 (or 5 when including alcohol) points with higher values reflecting closer adherence to the WCRF/AICR dietary recommendations. Cox regression analyses were performed to estimate multivariable-adjusted associations of the WCRF/AICR diet scores (per SD-increment) with risks of colorectal, colon, and rectal cancer. A similar approach was applied to the aMED indices to be able to compare model performances of both scores using AIC. Statistical analyses were conducted using Stata (version 15). Statistically significant results had a two-sided *P* value below 0.05.

## Results

Sex-specific median daily intakes of the aMEDr components among subcohort members are displayed in Table 1. As expected, median daily intakes of beneficial components increased with higher levels of MD adherence, whereas the opposite was observed for the intake of red and processed meats. Alcohol consumption was constant over the aMEDr categories in men, whereas in women slightly higher intakes were observed with closer adherence to the MD. Distributions of potential (colorectal) cancer risk factors (e.g. smoking status, BMI, and physical activity) over the aMEDr categories in the NLCS subcohort have been described in detail previously [45]. Comparing the highest to the lowest aMEDr category, subcohort members adhering more closely to the MD were less likely to smoke at baseline, had a lower BMI, and were more physically active. Generally, comparable levels of MD adherence were observed among colorectal cancer cases and subcohort members of both sexes, with mean aMEDr values of approximately 4 (Table 2). Considering other baseline characteristics, male and female colorectal cancer cases were more often former smokers compared to subcohort members, but less often current smokers (except female rectal cancer cases, Table 2). Additionally, levels of physical activity and alcohol consumption were higher in male, but lower in female, colorectal cancer cases. Furthermore, colon cancer cases were more likely to be highly educated than subcohort members (men only), whereas the opposite was observed for rectal cancer cases. Finally, colorectal cancer cases of both sexes more frequently reported a family history of this disease.

**Table 1:** Sex-specific median daily intakes of MD components (total and by aMEDr category) in subcohort members of the Netherlands Cohort Study

	Men				Women			
	All	aMEDr category			All	aMEDr category		
		0–3	4–5	6–8		0–3	4–5	6–8
	N = 2057	N = 855	N = 887	N = 315	N = 2027	N = 769	N = 901	N = 357
Vegetables (g)	207 (124)	177 (96)	222 (128)	266 (120)	219 (121)	179 (89)	228 (113)	272 (98)
Legumes (g)	6 (16)	0 (7)	9 (19)	13 (17)	5 (12)	0 (5)	6 (13)	11 (13)
Fruits (g)	157 (157)	120 (128)	167 (159)	230 (159)	209 (177)	161 (134)	227 (168)	284 (157)
Nuts (g)	3 (11)	1 (4)	5 (12)	9 (14)	2 (6)	0 (3)	2 (7)	5 (9)
Whole grains (g)	0 (10)	0 (0)	0 (16)	12 (41)	0 (13)	0 (0)	2 (15)	12 (23)
Fish (g)	11 (23)	6 (17)	14 (21)	22 (19)	9 (22)	3 (11)	12 (23)	20 (17)
Red and processed meats (g)	125 (63)	139 (64)	120 (61)	101 (51)	106 (61)	125 (62)	101 (56)	86 (45)
MUFA:SFA ratio	0.98 (0.24)	0.92 (0.21)	1.01 (0.23)	1.04 (0.14)	0.94 (0.21)	0.90 (0.19)	0.96 (0.21)	0.99 (0.20)
Alcohol (g)	10 (21)	10 (21)	10 (22)	9 (18)	2 (8)	1 (6)	2 (9)	3 (9)
Total energy (kcal)	2126 (648)	2125 (674)	2171 (653)	2057 (557)	1655 (516)	1670 (533)	1636 (475)	1664 (541)

*MD* Mediterranean diet, *aMEDr* alternate Mediterranean diet score without the alcohol component, *MUFA* monounsaturated fatty acids, *SFA* saturated fatty acids

Median (IQR) daily values in subcohort members are reported

**Table 2:** Sex-specific baseline characteristics of subcohort members and cases of colorectal cancer in the Netherlands Cohort Study

	Men				Women			
	Subcohort	Colorectal cancer cases			Subcohort	Colorectal cancer cases		
		All	Colon	Rectum		All	Colon	Rectum
	N = 2057	N = 2263	N = 1469	N = 549	N = 2027	N = 1703	N = 1275	N = 306
aMEDr	3.9 (1.6)	3.9 (1.6)	3.9 (1.6)	4.0 (1.5)	4.0 (1.6)	4.0 (1.6)	4.0 (1.6)	3.8 (1.6)
Age (years)^a^	61 (7)	61 (7)	62 (7)	61 (7)	61 (7)	62 (6)	62 (6)	62 (6)
Former cigarette smokers (%)	52.1	58.2	59.5	56.1	21.0	22.7	22.3	22.2
Current cigarette smokers (%)	35.1	29.9	28.1	33.3	21.3	19.9	19.4	22.2
Higher vocational education or university (%)	19.3	21.0	23.7	16.1	9.5	9.5	9.5	7.3
Alcohol consumption (g/day)^a^	9.7 (20.9)	11.3 (21.7)	10.6 (21.3)	11.4 (22.2)	1.6 (7.8)	1.4 (7.5)	1.5 (7.5)	1.3 (8.4)
Daily energy intake (kcal)	2162 (501)	2149 (483)	2135 (489)	2191 (473)	1687 (392)	1678 (374)	1673 (379)	1696 (339)
Body mass index (kg/m^2^)	24.9 (2.6)	25.2 (2.7)	25.2 (2.7)	25.1 (2.5)	25.0 (3.5)	25.0 (3.5)	25.0 (3.5)	25.0 (3.5)
Non-occupational physical activity (min/day)^a^	62.1 (67.1)	64.3 (64.3)	64.3 (64.3)	68.6 (60.0)	54.3 (52.9)	51.4 (53.6)	51.4 (53.6)	53.9 (50.0)
Family history of colorectal cancer (%)	5.3	9.1	9.5	7.3	6.0	9.9	10.4	8.8

*aMEDr* alternate Mediterranean diet score without the alcohol component

The % missing values in the total eligible population was < 5% for all variables included in this table. Mean (SD) values are reported unless otherwise specified

^a^Median (IQR) values are reported

Table 3 presents sex-specific and multivariable-adjusted associations of aMED, including and excluding alcohol, with risks of colorectal cancer and anatomical subsites. Age-adjusted associations can be found in Online Resource 1. Not all eligible subjects could be included in the Cox models because of missing information on covariates.

**Table 3:** Multivariable-adjusted associations of aMED (including and excluding alcohol) with colorectal cancer risk for men and women in the Netherlands Cohort Study

		Colorectum		Colon		Proximal colon		Distal colon		Rectum	
	PY_subcohort_	Cases	HR (95% CI)^a^	Cases	HR (95% CI)^a^	Cases	HR (95% CI)^a^	Cases	HR (95% CI)^a^	Cases	HR (95% CI)^a^
*Men*											
aMEDr											
0–3	11,788	779	1.00	507	1.00	232	1.00	256	1.00	178	1.00
4–5	12,448	873	1.07 (0.92–1.24)	566	1.07 (0.90–1.26)	244	1.02 (0.82–1.26)	307	1.13 (0.92–1.39)	218	1.17 (0.93–1.47)
6–8	4710	341	1.04 (0.85–1.28)	223	1.00 (0.80–1.26)	113	1.15 (0.87–1.52)	105	0.89 (0.67–1.19)	79	1.16 (0.85–1.59)
*P*_trend_			0.654		0.938		0.339		0.484		0.319
Continuous, per 2 pts	28,946	1993	1.04 (0.95–1.13)	1296	1.02 (0.92–1.12)	589	1.06 (0.94–1.20)	668	0.98 (0.87–1.11)	475	1.11 (0.97–1.27)
aMED^b^											
0–3	9167	613	1.00	396	1.00	183	1.00	198	1.00	143	1.00
4–5	12,891	883	1.04 (0.88–1.21)	576	1.05 (0.88–1.25)	256	1.02 (0.81–1.28)	304	1.09 (0.88–1.36)	214	1.07 (0.84–1.37)
6–9	6889	497	1.07 (0.89–1.28)	324	1.04 (0.84–1.28)	150	1.06 (0.82–1.38)	166	1.03 (0.79–1.33)	118	1.16 (0.87–1.53)
*P*_trend_			0.500		0.651		0.679		0.674		0.331
Continuous, per 2 pts	28,946	1993	1.03 (0.95–1.12)	1296	1.01 (0.92–1.11)	589	1.04 (0.92–1.17)	668	0.99 (0.88–1.11)	475	1.09 (0.96–1.23)
*Women*											
aMEDr											
0–3	12,149	619	1.00	460	1.00	274	1.00	173	1.00	117	1.00
4–5	14,963	656	0.86 (0.73–1.00)	498	0.87 (0.73–1.03)	284	0.84 (0.68–1.03)	201	0.92 (0.73–1.17)	113	0.81 (0.60–1.08)
6–8	6207	299	1.01 (0.82–1.23)	229	1.03 (0.83–1.29)	145	1.12 (0.87–1.45)	78	0.92 (0.68–1.24)	47	0.87 (0.60–1.27)
*P*_trend_			0.941		0.755		0.377		0.575		0.462
Continuous, per 2 pts	33,318	1574	0.97 (0.88–1.07)	1187	0.99 (0.89–1.10)	703	1.02 (0.90–1.15)	452	0.96 (0.83–1.10)	277	0.91 (0.76–1.08)
aMED^b^											
0–3	10,637	543	1.00	406	1.00	240	1.00	155	1.00	101	1.00
4–5	14,551	640	0.86 (0.73–1.01)	478	0.85 (0.72–1.02)	281	0.85 (0.69–1.05)	183	0.86 (0.67–1.10)	114	0.83 (0.62–1.12)
6–9	8130	391	0.97 (0.80–1.18)	303	1.01 (0.82–1.24)	182	1.03 (0.81–1.32)	114	0.99 (0.75–1.31)	62	0.86 (0.60–1.23)
*P*_trend_			0.960		0.744		0.637		0.917		0.453
Continuous, per 2 pts	33,318	1574	0.97 (0.89–1.06)	1187	0.98 (0.89–1.08)	703	0.99 (0.88–1.12)	452	0.98 (0.86–1.12)	277	0.91 (0.77–1.07)

*aMED* alternate Mediterranean diet score, *PY*_*subcohort*_ person-years in the subcohort, *aMEDr* alternate Mediterranean diet score without the alcohol component

^a^Adjusted for age at baseline (years), cigarette smoking status (never, former, current), cigarette smoking frequency (cigarettes smoked per day, centered), cigarette smoking duration (years, centered), body mass index (kg/m^2^), alcohol consumption (0, > 0 to < 5, ≥ 5 to < 15, ≥ 15 to < 30, ≥ 30 g/day), daily energy intake (kcal), highest level of education (primary school or lower vocational, secondary school or medium vocational, higher vocational or university), non-occupational physical activity (≤ 30, > 30 to ≤ 60, > 60 to ≤ 90, > 90 min/day), and family history of colorectal cancer (no, yes)

^b^Not adjusted for alcohol consumption

In men, aMEDr was not significantly associated with colorectal cancer risk in categorical and continuous analyses [HR_per two-point increment_ (95% CI) 1.04 (0.95–1.13)] (Table 3). Subsite-specific HR estimates per two-point increment in aMEDr (all not statistically significant) ranged from 0.98 for distal colon cancer to 1.11 for rectal cancer and did not significantly differ [*P*_heterogeneity_: 0.566 (proximal vs. distal colon) and 0.518 (colon vs. rectum)]. Similar to men, no significant association was observed between aMEDr and colorectal cancer risk in women [HR_per two-point increment_ (95% CI) 0.97 (0.88–1.07)] (Table 3). However, middle vs. low aMEDr values were associated with a borderline significantly reduced colorectal cancer risk [HR (95% CI) 0.86 (0.73–1.00)]. Though subsite-specific associations were all not statistically significant and there was no evidence of heterogeneity [*P*_heterogeneity_: 0.690 (proximal vs. distal colon) and 0.194 (colon vs. rectum)], results suggested a weak inverse association between aMEDr and rectal cancer risk in women [HR_per two-point increment_ (95% CI) 0.91 (0.76–1.08)]. Comparable results were obtained for the original aMED including alcohol in both men and women (Table 3). For colorectal cancer risk, inclusion of alcohol in the MD score resulted in a worse model fit.

Associations of aMEDr with colorectal cancer risk in women differed statistically significantly across strata of smoking status (*P*_interaction_ = 0.015, Table 4). In female ex-smokers, increasing aMEDr values were associated with a significantly reduced colorectal cancer risk [HR_per two-point increment_ (95% CI) 0.78 (0.63–0.98)]. In contrast, a positive association was suggested in female current smokers, with a significant positive trend over the aMEDr categories (*P*_trend_ = 0.04, data not shown). Finally, there was no evidence of an association in women who had never smoked. No significant interactions were observed between aMEDr and other potential colorectal cancer risk factors (alcohol consumption, BMI, educational level, and family history of colorectal cancer) in men and women, or smoking status in men (Table 4). Simultaneous inclusion of all aMEDr components as dichotomous variables in multivariable-adjusted models showed that none of the individual components was significantly associated with colorectal cancer risk (data not shown). Associations were comparable after exclusion of the first 2 years of follow-up and did not significantly differ across the three follow-up periods (data not shown).

**Table 4:** Sex-specific and multivariable-adjusted associations of aMEDr (per two-point increment) with colorectal cancer risk for various subgroups in the Netherlands Cohort Study

	Colorectal cancer			
	Men		Women	
	Cases	HR (95% CI)^a,b^	Cases	HR (95% CI)^a,b^
Cigarette smoking status^c^				
Never	256	1.11 (0.87–1.41)	915	1.00 (0.88–1.13)
Former	1184	1.03 (0.92–1.15)	350	0.78 (0.63–0.98)
Current	553	1.02 (0.85–1.22)	309	1.21 (0.96–1.51)
*P*_interaction_^d^		0.714		0.015
Alcohol consumption^e^				
0 g/day	235	1.14 (0.90–1.46)	489	1.04 (0.86–1.25)
> 0 to < 15.0 g/day	934	1.05 (0.92–1.19)	865	0.97 (0.86–1.10)
≥ 15.0 g/day	824	0.98 (0.85–1.13)	220	0.88 (0.66–1.17)
*P*_interaction_^d^		0.731		0.539
Body mass index^f^				
≥ 18.5 to < 25.0 kg/m^2^	970	1.04 (0.92–1.17)	848	1.03 (0.90–1.17)
≥ 25.0 kg/m^2^	1018	1.05 (0.92–1.19)	707	0.90 (0.78–1.04)
*P*_interaction_^d^		0.876		0.232
Highest level of education^g^				
Primary school or lower vocational	863	0.97 (0.84–1.12)	841	0.95 (0.84–1.08)
Secondary school or medium vocational	706	1.04 (0.90–1.19)	579	1.00 (0.84–1.19)
Higher vocational or university	424	1.21 (0.98–1.50)	154	1.08 (0.79–1.47)
*P*_interaction_^d^		0.133		0.920
Family history of colorectal cancer^h^				
No	1811	1.06 (0.97–1.16)	1412	0.99 (0.89–1.09)
Yes	182	0.82 (0.55–1.23)	162	0.90 (0.59–1.39)
*P*_interaction_^d^		0.204		0.423

*aMEDr* alternate Mediterranean diet score without the alcohol component

^a^All HRs were estimated per two-point increment in aMEDr

^b^Adjusted for age at baseline (years), cigarette smoking status (never, former, current), cigarette smoking frequency (cigarettes smoked per day, centered), cigarette smoking duration (years, centered), body mass index (kg/m^2^), alcohol consumption (0, > 0 to < 5, ≥ 5 to < 15, ≥ 15 to < 30, ≥ 30 g/day), daily energy intake (kcal), highest level of education (primary school or lower vocational, secondary school or medium vocational, higher vocational or university), non-occupational physical activity (≤ 30, > 30 to ≤ 60, > 60 to ≤ 90, > 90 min/day), and family history of colorectal cancer (no, yes)

^c^Not adjusted for cigarette smoking status

^d^*P* values for interaction were obtained by testing the statistical significance of interaction terms between aMEDr and the stratifying covariates in multivariable-adjusted models

^e^Not adjusted for alcohol consumption

^f^Not adjusted for body mass index

^g^Not adjusted for highest level of education

^h^Not adjusted for family history of colorectal cancer

Like the population-dependent aMED indices, the absolute WCRF/AICR diet scores (including and excluding alcohol) were not significantly associated with risks of colorectal, colon, and rectal cancer in men and women (Table 5). Performances of models containing aMED indices and WCRF/AICR diet scores were mostly comparable.

**Table 5:** Sex-specific and multivariable-adjusted associations of the absolute WCRF/AICR diet score and aMED (per SD-increment) with colorectal cancer risk in the Netherlands Cohort Study

	Men			Women		
	Colorectum	Colon	Rectum	Colorectum	Colon	Rectum
	HR_SD_ (95% CI)^a,b^	HR_SD_ (95% CI)^a,b^	HR_SD_ (95% CI)^a,b^	HR_SD_ (95% CI)^a,b^	HR_SD_ (95% CI)^a,b^	HR_SD_ (95% CI)^a,b^
PY_subcohort_/cases^c^	28,304/1933	28,304/1256	28,304/462	32,678/1545	32,678/1166	32,678/270
Excluding alcohol						
WCRF/AICR diet score^d^	0.99 (0.93–1.07)	0.97 (0.90–1.05)	1.03 (0.92–1.14)	0.99 (0.92–1.07)	1.01 (0.93–1.10)	0.97 (0.85–1.12)
aMEDr	1.03 (0.96–1.10)	1.01 (0.93–1.09)	1.09 (0.98–1.21)	0.98 (0.91–1.05)	0.99 (0.91–1.07)	0.93 (0.81–1.07)
Including alcohol						
WCRF/AICR diet score^d,e^	0.95 (0.88–1.02)	0.96 (0.88–1.04)	0.93 (0.83–1.04)	1.00 (0.92–1.07)	1.01 (0.93–1.10)	0.96 (0.83–1.11)
aMED^e^	1.02 (0.96–1.10)	1.01 (0.93–1.09)	1.07 (0.97–1.19)	0.97 (0.90–1.05)	0.98 (0.90–1.07)	0.92 (0.80–1.06)

*WCRF/AICR* World Cancer Research Fund/American Institute for Cancer Research, *aMED* alternate Mediterranean diet score, *PY*_*subcohort*_ person-years in the subcohort, *aMEDr* alternate Mediterranean diet score without the alcohol component

^a^HRs were estimated per SD-increment in the scores

^b^Adjusted for age at baseline (years), cigarette smoking status (never, former, current), cigarette smoking frequency (cigarettes smoked per day, centered), cigarette smoking duration (years, centered), body mass index (kg/m^2^), alcohol consumption (0, > 0 to < 5, ≥ 5 to < 15, ≥ 15 to < 30, ≥ 30 g/day), daily energy intake (kcal), highest level of education (primary school or lower vocational, secondary school or medium vocational, higher vocational or university), non-occupational physical activity (≤ 30, > 30 to ≤ 60, > 60 to ≤ 90, > 90 min/day), and family history of colorectal cancer (no, yes)

^c^A lower number of subjects could be included in these analyses as a result of missing values for salt intake

^d^Score based on the WCRF/AICR dietary recommendations to prevent cancer issued in 2007

^e^Not adjusted for alcohol consumption

## Discussion

In this prospective cohort study, a priori defined MD adherence, assessed by aMEDr, was not significantly associated with colorectal cancer risk. Associations were absent for all investigated anatomical subsites and in both men and women. The association between aMEDr and colorectal cancer risk in women was significantly modified by smoking status (*P*_interaction_ = 0.015). A significant inverse association was observed in female ex-smokers, whereas a positive association was suggested in female current smokers. For colorectal cancer risk, the best model performance was obtained when alcohol intake was not included in the MD score.

Various prospective cohorts have investigated the relation of a priori defined MD adherence with colorectal cancer risk and indicated disparate associations for men and women. In men, higher MD adherence has fairly consistently been associated with a reduced colorectal cancer risk (but not always significant) [9, 10, 13, 16, 17, 20]. For example, in male participants of the Multiethnic Cohort Study (MEC), the National Institutes of Health (NIH)-AARP Diet and Health Study, and the Health Professionals Follow-up Study (HPFS), statistically significant HR estimates of 0.84, 0.72, and 0.80, respectively, were obtained when comparing high to low aMED values [13, 16, 20]. Furthermore, high compared to low MD adherence (modified Mediterranean diet score) was associated with a non-significantly reduced colorectal cancer risk in the male part of the EPIC cohort [HR (95% CI) 0.89 (0.76–1.04)] [10]. With some exceptions [9, 12], studies in women did not support the presence of an inverse association between MD adherence and colorectal cancer risk [10, 13, 15–18, 20]. For comparison, non-significant HR estimates of 0.96 (MEC), 0.89 (NIH-AARP), 0.99 (Nurses’ Health Study, NHS), and 0.88 (EPIC), were reported for high vs. low MD adherence in female participants of the abovementioned studies [10, 13, 16, 20]. In the present analysis of the NLCS, a priori defined MD adherence was not associated with a significantly decreased risk of colorectal cancer in both sexes. Similar to our analysis, the majority of the previously conducted studies used aMED (variants) to assess MD adherence. However, the particular food items included in the aMED components may have differed between studies, which could (partly) explain the contrasting results that we observed for men in our cohort. Additionally, the more homogenous nature of the NLCS study population may have resulted in relatively small contrasts in absolute food intakes between subjects in the highest and lowest adherence categories making it more difficult to detect potentially beneficial effects of the MD on health outcomes. Median daily intakes among male NLCS subcohort members in the highest and lowest aMEDr categories were for example 266 g and 177 g for vegetables, 230 g and 120 g for fruits, and 101 g and 139 g for red and processed meats, respectively. In male participants of the HPFS [20], the mean numbers of servings per day in the highest and lowest aMED quintiles were 4.9 and 2.0 for vegetables, 2.6 and 0.8 for fruits, and 0.7 and 1.2 for red and processed meats. We calculated ratios comparing median/mean intakes in the highest and lowest MD categories. The ratios showed clearly higher contrasts in intakes of vegetables and fruits in the HPFS [vegetables: 1.5 (NLCS) vs. 2.5 (HPFS), fruits: 1.9 (NLCS) vs. 3.3 (HPFS)]. The contrast in the intake of red and processed meats was comparable in both cohorts [0.7 (NLCS) vs. 0.6 (HPFS)]. We were forced to compare median daily intakes in the NLCS with mean numbers of servings per day in the HPFS, because there were no other data available. Despite our relatively homogeneous study population, we previously detected significant inverse associations between aMEDr and risks of esophageal squamous cell carcinoma, gastric cardia adenocarcinoma (GCA), and gastric non-cardia adenocarcinoma (GNCA) in men in the NLCS [46], suggesting sufficient contrast. In female NLCS participants, associations of aMEDr with risks of GCA and GNCA were also inverse, but did not reach statistical significance.

None of the aMEDr components was individually associated with colorectal cancer risk in the present study. Possibly, the individual effects of the aMEDr components were too weak to be detected. Combining these components into a dietary pattern score increases the likelihood that the potentially weak individual effects are being detected. Furthermore, by investigating the effect of a dietary pattern, one allows for synergistic or antagonistic interactions between the dietary components, and solves confounding and collinearity problems associated with the analysis of single food groups. Finally, the contrast within the study population in terms of overall healthiness of the diet is possibly increased when considering the MD as a whole, which increases the chance of detecting true effects, if present [31, 47, 48].

The potentially distinct etiological backgrounds of tumors arising in the proximal colon, distal colon, and rectum and varying exposures to (carcinogens in) fecal matter across subsites may cause heterogeneous susceptibilities to (lifestyle) risk factors [21, 49]. Subsite-specific analyses in the NIH-AARP and HPFS cohorts demonstrated that the inverse association between MD adherence and colorectal cancer risk in men was particularly pronounced for distal colon cancer and rectal cancer [13, 20]. However, associations did not seem to differ across the subsites in men in our study. Additionally, there was no clear evidence for heterogeneity across the anatomical subsites in women, both in our cohort and in most previous studies [13, 15, 20].

In women in our cohort, associations between MD adherence and colorectal cancer risk significantly differed across strata of smoking status, with oppositely directed associations being observed in former smokers (inverse) and current smokers (positive). Smoking status did not significantly interact with MD adherence in female participants of the NHS [20]. However, this study did not differentiate between former and current smokers. A possible explanation for the interaction with smoking status that we observed is chance, considering the large number of tests performed. We recommend that the potentially modifying role of smoking status in the association between MD adherence and colorectal cancer risk, as well as underlying mechanisms, are investigated in future studies. Preferably, these studies should be performed separately for men and women, and distinguish between former and current smokers.

Colorectal cancers usually develop slowly over the course of 10–15 years [3], making the prospective design and long duration of follow-up major strengths of the present study. The large number of cases diagnosed during follow-up facilitated the performance of sex-specific analyses for cancers of the colorectum, colon, proximal colon, distal colon, and rectum with acceptable statistical power, while adjusting for relevant confounders. Additionally, associations were estimated within strata of colorectal cancer risk factors, separately for men and women. Since the national population screening program for colorectal cancer in the Netherlands started after the end of follow-up of our study [50], it could not have influenced the results. Despite the high quality of the dietary information, possible measurement error may have attenuated associations. Another limitation is the single measurement of diet and lifestyle factors at baseline. Changes in diet and lifestyle factors during follow-up may have led to non-differential misclassification and attenuated associations. However, the baseline assessment of the NLCS-FFQ has been shown to be capable of ranking subjects according to their nutrient intakes relatively well for over at least 5 years [28]. Furthermore, associations between aMEDr and colorectal cancer risk were largely similar, and did not significantly differ, across the three periods of follow-up (≤ 2, > 2 to ≤ 10, and > 10 years). Residual confounding by unmeasured factors also cannot be excluded. Lastly, aMEDr assesses the relative level of MD adherence using population-based cut-offs. Therefore, subjects with high scores do not necessarily adhere closely to a traditional MD, particularly in non-Mediterranean study populations. Comparison of diets of the Netherlands and Greece using previously reported intake data from the EPIC cohort [51] showed that mean daily intakes of food groups typically consumed in large amounts in the MD, such as vegetables, fruits, and legumes, were lower in participants of the Dutch EPIC cohorts (EPIC-NL) compared to participants of the Greek EPIC cohort (EPIC-Greece). Mean daily intakes of vegetables, fruits, and legumes among men were 131 g, 156 g, and 6 g in EPIC-NL and 269 g, 234 g, and 33 g in EPIC-Greece, respectively. Among female participants of EPIC-NL and EPIC-Greece, mean daily intakes were 128 g and 211 g for vegetables, 183 g and 218 g for fruits, and 4 g and 21 g for legumes, respectively. As expected, meat consumption was higher in Dutch subjects [EPIC-NL: 141 g (men) and 80 g (women), EPIC-Greece: 68 g (men) and 35 g (women)] [51]. Regardless of its use of population-based cut-offs, the model fit of aMEDr was generally comparable to that of the absolute WCRF/AICR diet score in our study.

In conclusion, results of this large prospective cohort study do not support the hypothesis that higher MD adherence is associated with a reduced risk of colorectal cancer. MD adherence was not significantly associated with the risk of any of the colorectal cancer subsites in both men and women.

## Electronic supplementary material

Below is the link to the electronic supplementary material.

Supplementary material 1 (PDF 528 kb)
