# Supplementary material for: Mediterranean diet adherence and risk of colorectal cancer: the prospective Netherlands Cohort Study
Source: Eur J Epidemiol. 2019 Sep 7;35(1):25–35. doi: 10.1007/s10654-019-00549-8 (PMC7058569; doi:10.1007/s10654-019-00549-8)
Supplement: Supplementary file 1 — Supplementary material 1 (PDF 528 kb) [file 10654_2019_549_MOESM1_ESM.pdf]

Mediterranean diet adherence and risk of colorectal cancer: the prospective Netherlands Cohort Study

European Journal of Epidemiology

Maya Schulpen, Piet A. van den Brandt

Corresponding author: Piet A. van den Brandt, Department of Epidemiology, Maastricht University Medical Centre, P.O. Box 616, 6200 MD Maastricht, the Netherlands. E-mail: pa.vandenbrandt@maastrichtuniversity.nl

Online Resource 1 Age-adjusted associations of aMEDr with colorectal cancer risk for men and women in the Netherlands Cohort Study

		Colorectum		Colon		Proximal colon		Distal colon		Rectum	
	$PY_{subcohort}$	Cases	HR (95% CI)[a]	Cases	HR (95% CI)[a]	Cases	HR (95% CI)[a]	Cases	HR (95% CI)[a]	Cases	HR (95% CI)[a]
Men											
aMEDr											
0-3	11788	779	1.00	507	1.00	232	1.00	256	1.00	178	1.00
4-5	12448	873	1.06 (0.92 - 1.23)	566	1.06 (0.90 - 1.24)	244	1.00 (0.81 - 1.23)	307	1.13 (0.93 - 1.38)	218	1.16 (0.93 - 1.45)
6-8	4710	341	1.07 (0.88 - 1.29)	223	1.07 (0.86 - 1.32)	113	1.17 (0.89 - 1.53)	105	1.00 (0.76 - 1.31)	79	1.09 (0.81 - 1.48)
P_{trend}			0.497		0.544		0.265		0.927		0.513
Continuous, per 2 pts	28946	1993	1.04 (0.96 - 1.13)	1296	1.04 (0.95 - 1.14)	589	1.07 (0.95 - 1.20)	668	1.03 (0.92 - 1.15)	475	1.08 (0.96 - 1.23)
Women											
aMEDr											
0-3	12149	619	1.00	460	1.00	274	1.00	173	1.00	117	1.00
4-5	14963	656	0.87 (0.75 - 1.02)	498	0.89 (0.76 - 1.05)	284	0.86 (0.70 - 1.04)	201	0.95 (0.76 - 1.19)	113	0.79 (0.60 - 1.05)
6-8	6207	299	0.97 (0.80 - 1.18)	229	1.00 (0.81 - 1.24)	145	1.08 (0.84 - 1.38)	78	0.90 (0.66 - 1.21)	47	0.81 (0.56 - 1.16)
P_{trend}			0.781		0.969		0.547		0.474		0.244
Continuous, per 2 pts	33318	1574	0.96 (0.88 - 1.05)	1187	0.98 (0.89 - 1.08)	703	1.01 (0.89 - 1.13)	452	0.96 (0.84 - 1.10)	277	0.88 (0.74 - 1.04)

aMEDr alternate Mediterranean diet score without the alcohol component, *$PY_{subcohort}$* person-years in the subcohort

[a]Adjusted for age at baseline (years)
